# Atypical Hemolytic Uremic Syndrome Presenting as Pre-eclampsia in a 24-year-old Woman with Chronic Kidney Disease: Pathogenesis and Genetics

**DOI:** 10.7759/cureus.3358

**Published:** 2018-09-25

**Authors:** Junior Kalambay, Rehana Zaman, Mohammad Zaman

**Affiliations:** 1 Internal Medicine, West Side Medical Center, New York, USA; 2 Internal Medicine, Mount Sinai St. Lukes and West Hospital Center, New York, USA; 3 Internal Medicine, Brookdale University Hospital, New York, USA

**Keywords:** atypical hemolytic uremic syndrome, microangiopathic hemolytic anemia, alternative complement pathway, adamst13, genetic defect, incomplete penetrance, familial, sporadic, ecluzimab, pathogenesis

## Abstract

Atypical hemolytic uremic syndrome (aHUS) is a kidney disorder that is frequently unrecognized during its progression, and misdiagnosed with more common etiologies of microangiopathic hemolytic anemia (MAHA): hemolytic uremic syndrome, disseminated intravascular coagulation, and thrombotic thrombocytopenic purpura (TTP). During pregnancy, the diagnosis of aHUS is furthermore challenging. The clinical presentation of aHUS may mimic pre-eclampsia as it occurred to the patient described in the case report. However, the persistence of thrombocytopenia in the patient after dilatation of the cervix and surgical evacuation of the contents of the uterus has led to consider aHUS. The pathogenesis of aHUS provides clues to understanding the insidious progression and the variability of clinical presentations of the disease. aHUS is primarily a kidney disorder that results from genetic defects of the alternative complement pathway (AP). Consequently, Eculizumab, a monoclonal antibody that targets the AP, induced remission in the patient. A single gene defect of the AP cannot cause the clinical manifestation of aHUS alone. Most of aHUS patients have a combination of mutation, haplotype, and single nucleotide polymorphism. Often, an identifiable environmental factor or a physiological change triggers the onset of the disease. We report the first case of aHUS in a pregnant woman with chronic kidney disease.

## Introduction

Atypical hemolytic uremic syndrome (aHUS) is an extremely rare etiology of microangiopathic hemolytic anemia (MAHA). In common, disorders with MAHA affect endothelial cells of small vessels and cause mechanical destructions of red blood cells. Other MAHA etiologies include hemolytic uremic syndrome, disseminated intravascular coagulations and thrombotic thrombocytopenic purpura (TTP). aHUS is 10 times less common than HUS, and only two cases of aHUS per million persons are reported each year in the United States [1,2].

Unlike other etiologies of MAHA that develop in vascular system, aHUS is primarily a renal disorder. aHUS involves genetic defects of regulator genes of the alternative complement pathway (AP) and results in the overactivation of the alternative pathway of the complement system in the kidneys. Together, direct injuries from membrane attack complex of the complement (MAC) and injuries from endothelial dysfunction cause microvascular thrombosis, ischemia, glomerular dysfunction and acute renal failure [2]. Extrarenal manifestations of aHUS are often due to an increased vascular permeability in organs secondary to the circulating anaphylatoxins C3a and C5a produced in the kidneys [3].

aHUS can be familial or sporadic. Up to 20% of aHUS cases are familial. Familial aHUS has a poor prognosis with a rate of end-stage renal disease or death of 50% to 90%. Half cases of sporadic aHUS are idiopathic, the other half cases of aHUS patients have an identifiable trigger such as a pregnancy, an infection with human immunodeficiency virus, an organ transplant, or a malignancy. These events are considered to enhance AP activation [4-7].

## Case presentation

A 24-year-old G2 P0 patient with history of chronic hypertension and chronic unspecified kidney disease diagnosed in infancy and followed by a nephrologist was admitted for one-day history of epigastric and right upper quadrant pain. The pain was sharp, intermittent with an intensity of 9/10. On admission day, the pain increased two hours after eating spicy tacos. She denies any exacerbating or alleviating factor. The patient also vomited six times after eating.

The patient also reported painful uterine contraction. On physical exam, the patient was hemodynamically stable. The vaginal exam revealed 3–4 cm dilated and effaced cervix.

On admission, laboratory results were consistent with microangiopathic hemolytic anemia (severe thrombocytopenia, anemia, worsening kidney function) (Table 1).

**Table 1:** Laboratory results on admission. WBC: White blood cell; RBC: Red blood cell count; MCV: Mean corpuscular volume; MCH: Mean corpuscular hemoglobin; MCHC: Mean corpuscular hemoglobin concentration; RDW: Red blood cell distribution width; MPV: Mean platelet volume; PT: Prothrombin time; INR: International normalized ratio; AST: Aspartate aminotransferase; ALT: Alanine aminotransferase; A/G: Albumin/globulin ratio; GFR: Glomerular filtration rate.

Cell Blood Count		Complete Metabolic Panel	
WBC	13.5	Sodium	133
Hemoglobin	9.4	Potassium	5.1
Hematocrit	27.4	Chloride	111
Platelet count	52	CO2	11
RBC	3.09	Urea Nitrogen	88
MCV	88.6	Creatinine	5.58
MCH	30.5	Glucose	96
MCHC	34.4	Est GFR	9
RDW	12.3	Anion Gap	11
MPV	8.9	Calcium	7.8
Coagulation Studies		Protein, Total	5.5
PT	13.9	Albumin	2.9
INR	1.1	A/G ratio	1.1
Antiphospholipid Antibody-Panel-Status		Bilirubin, Total	0.4
Antiphospholipid IgG	6	Alkaline Phosphatase	82
Antiphospholipid IgM	5	AST	209
		ALT	149

The delivery team was consulted for dilation and evacuation for a possible pre-eclampsia/HELLP syndrome. Failure of thrombocytopenia to improve after dilation and evacuation led to consider TTP and aHUS. TTP is treated with plasma exchange, and aHUS is treated with anti-complement therapy. TTP was considered and treated presumptively given the severity of its complication: intracranial bleeding. Creatinine level was monitored to assess the response to treatment. A lack of improvement would change the presumptive diagnosis of TTP to aHUS. ADAMTS 13 level was sent prior to any transfusion. ADAMTS level was low at 44% favoring aHUS. The patient entered in remission when she was treated with Eculizumab.

Imaging and genetic tests

**Figure 1:**
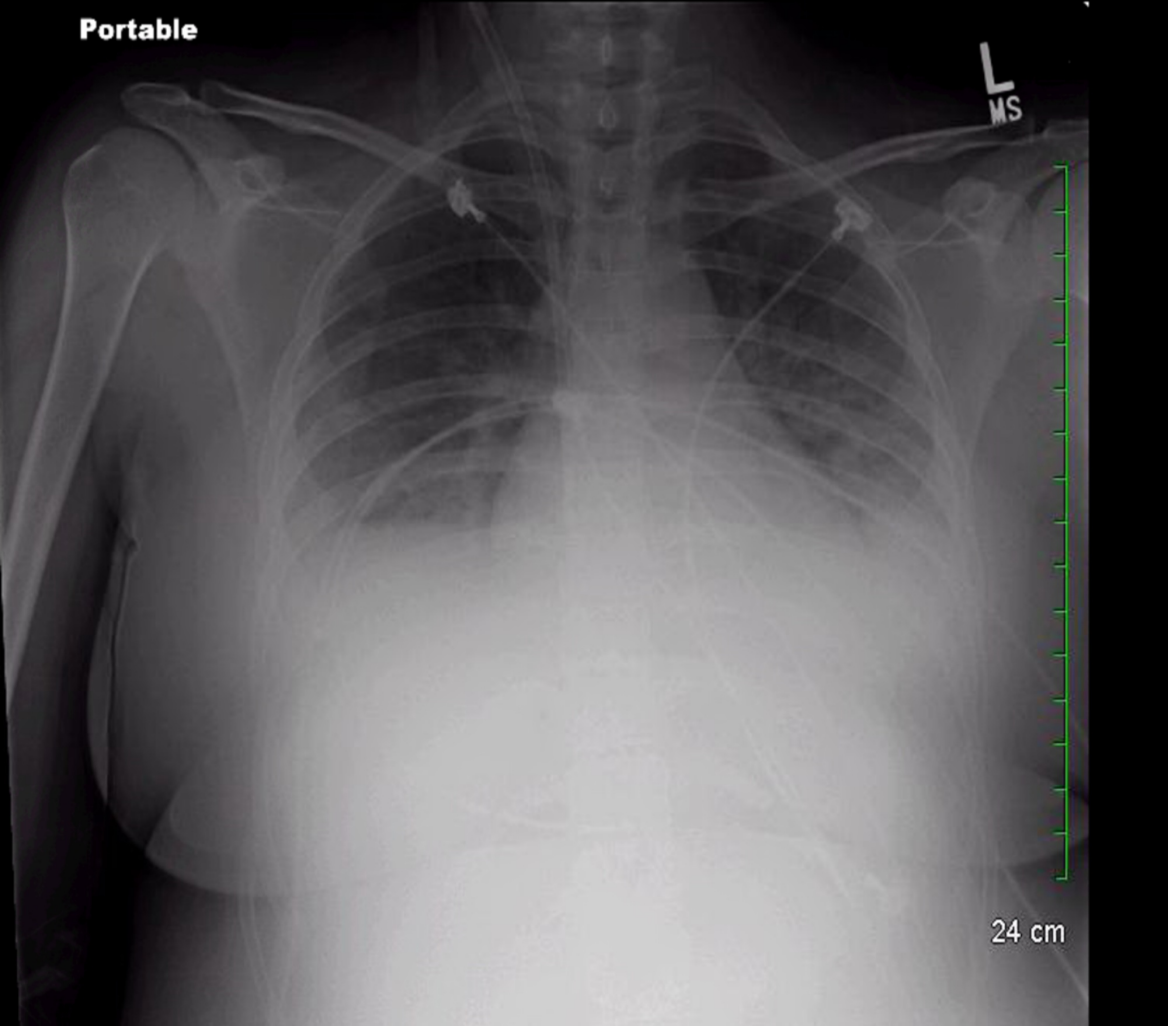
Chest X-ray. Cardiac silhouette is normal in size. Bilateral basilar opacities likely represent moderate pleural effusions.

Chest X-ray showed basilar opacities, likely moderate pleural effusions (Figure 1).

The abdominal sonogram demonstrated an atrophic right kidney with increased echogenicity, slightly prominent tortuous veins at the splenic hilum which may represent focal perisplenic varices.

Genetic testing

DNA sequencing and multiplex ligation-dependent probe amplification (MLPA) analysis did not identify any significant variant of the genes tested: CPH, CFI, MCP, THBD, VFB, C3, DGKE, ADAMTS 13, C4BPA, C4BPB, LMNA, CFTHR1, CFHR3. Genetic variants identify only 50–60% of aHUS cases.

## Discussion

Genetics of the complement system regulation: the basis of aHUS pathogenesis, incomplete penetrance, and prognosis

The activation of the AP of the complement system is tightly regulated, and defects of the AP genes and regulatory genes are involved in the development of aHUS. The spontaneous hydrolysis of C3 into C3H20 in the plasma initiates the AP. C3H20 generates C3b that binds to the surface of plasma membranes. On the surface of plasma membranes, C3b interacts with Factor B. Factor D will later on cleave Factor B and conclude the formation of the C3 convertase of the AP (C3bBb). C3 convertase cleaves C3 into C3a and C3b. The newly generated C3b can interact with Factor B and create an amplification loop that will activate the AP until all the complement components are consumed. Complement factor I (CFI) inactivates C3b into C3bi and prevents C3b interaction with Factor B. The reaction of CFI is possible only if the Complement Factor H (CFH) is bound to both C3b and the host cell, and the co-factor membrane complement protein (MCP) is functional. CFH has a discriminative property. It can bind only to host cells and not to pathogens. CFH distinguishes host cells from pathogens and prevents host cells from undergoing complement-mediated cells lysis. Thrombomodulin (TM) enhances the activity of CFI. Complement regulatory proteins prevent the consumption of all complement substrates and restrict the activation of the complement system on the surface of microorganisms.

Loss of function mutations of regulatory genes

**Figure 2:**
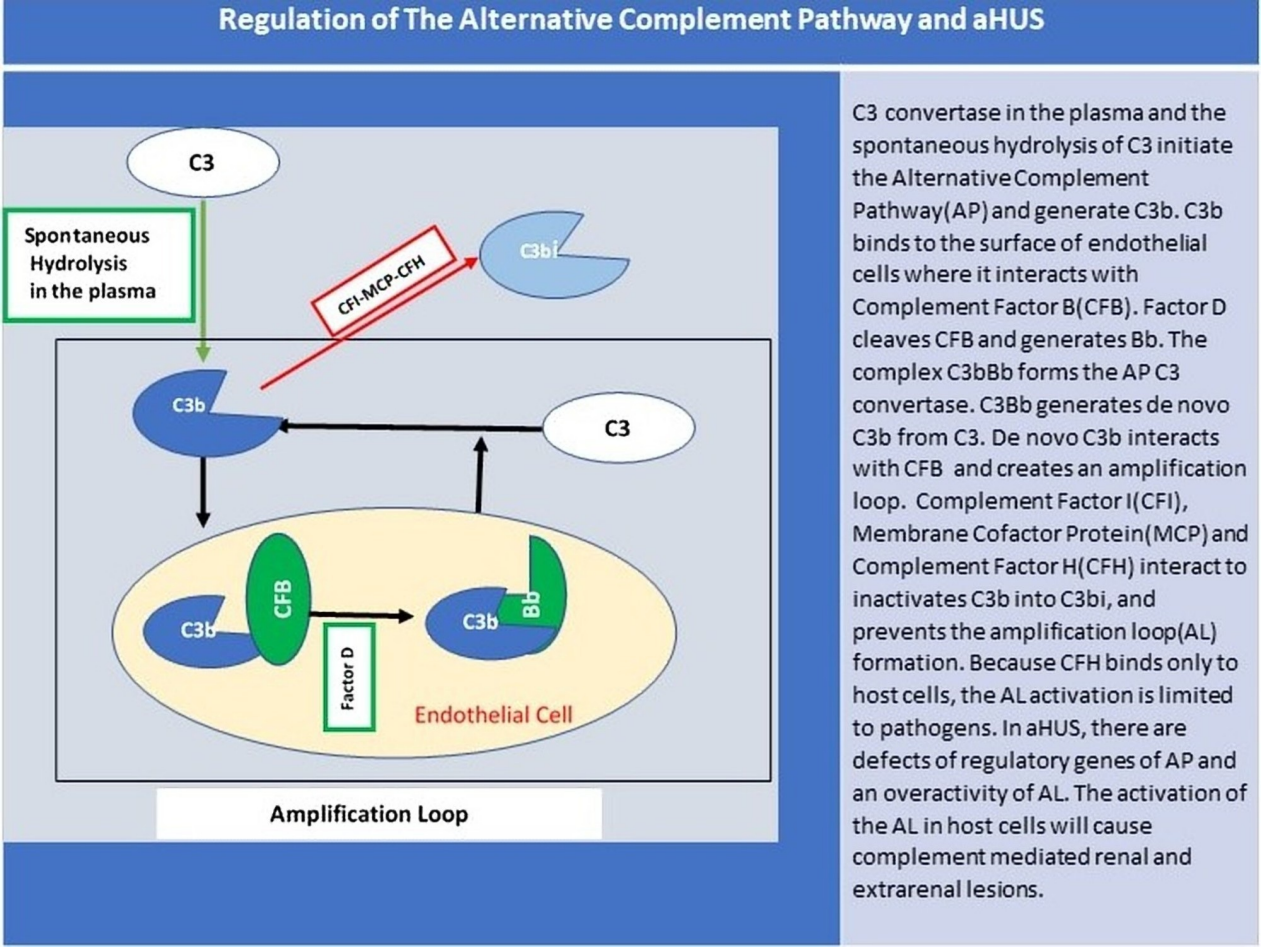
Regulation of the alternative complement pathway and aHUS. aHUS: Atypical hemolytic uremic syndrome

CFH, MCP, TM or CFI and increased function mutations of C3b and Factor B have been all identified in the pathogenesis of HUS [4,7]. About 6 to 10% of aHUS patients have an anti-CFH antibody that prevents a proper binding of CFH to C3b. Interestingly, anti-CFH antibodies develop in young children who are homozygous for the deletion of CFHR1 and CFHR3 genes (Figure 2) [8].

The alteration of a single gene alone cannot cause aHUS. In a study where patients and their relatives were screened for mutation and polymorphism for CFH, MCP, and CFI genes, patients did not develop aHUS unless they had also a combination of mutations, haplotypes and single nucleotides polymorphism (SNP). In a patient with MCP mutation, for example, MCPggaac haplotype increases two-fold the risk of aHUS compared with control. MCPggaac haplotype contains two SNPs in the MCP promoter which decreases the transcription of MCP [9,10].

The genetic origin of aHUS also explains the incomplete penetrance. For mutation involving CFH, CFI, MCP, and CFB, the penetrance is ∼50%. Even when many altered genes are associated with the same patient, aHUS develops only after the occurrence of certain environmental factors. Pregnancy, HIV, and oral contraceptive pills have been reported to trigger aHUS in genetically predisposed patients [4].

Genetic predispositions also determine the prognosis after the first episode and after renal transplantation. MCP patients have a better prognosis with a complete remission rate of 80 to 90%. CFH, CFI, C3 mutation and anti-CFH antibody are associated with poor outcome in the first episode. Renal transplantation is favorable for patients with MCP mutations as well, whereas it has a poor success rate for patients with CFI and CFH mutations [4,11,12].

## Conclusions

aHUS is a disorder of the alternative complement pathway. Genetic defects of components that regulate the AP lead to unopposed activation of AP in the kidneys. Local endothelial cells lesions and MAC formation, and circulatory C3a and C5a explain renal and extrarenal manifestations, respectively. Clinically, aHUS may be challenging to distinguish from pre-eclampsia in a pregnant patient because they both share thrombocytopenia, and a pregnancy can trigger both diseases. It is not clear whether chronic kidney disease in the patient increased the likelihood of pre-eclampsia-like presentation. The initial management should favor urgency and consider pre-eclampsia. The lack of response to the pre-eclampsia treatment and the ADAMST 13 level will readjust the presumptive diagnosis to either HUS or aHUS. We report the first case of aHUS presenting as pre-eclampsia in a pregnant woman with severe kidney disease.
